# Association between dyslipidaemia knowledge & lipid testing practice among adults, a community-based study

**DOI:** 10.1371/journal.pone.0306428

**Published:** 2024-07-30

**Authors:** Haranee Paramalingam, Aqil M. Daher, Sumaira Hussain

**Affiliations:** 1 School of Postgraduate Studies, IMU University, Kuala Lumpur, Malaysia; 2 Department of Public Health & Community Medicine, School of Medicine, IMU University, Kuala Lumpur, Malaysia; Muhimbili University of Health and Allied Sciences School of Medicine, UNITED REPUBLIC OF TANZANIA

## Abstract

Various factors have been described in the literature to explain the tendency of an individual to undergo medical screenings. This study aimed to assess the association between the level of knowledge about dyslipidaemia and the frequency of lipid testing, as well as the potential impact of predisposing, enabling, and need factors on the uptake of blood lipid screening. This study was a cross-sectional survey involving 314 participants who were Malaysian residents of Taman Selatan, Klang, aged 30 and above. The study utilized a 42-item paper-based bilingual questionnaire to evaluate the predisposing, enabling, and need factors that could potentially influence the practice of lipid testing. Out of the 314 residents approached, 271 responded to the questionnaire yielding a response rate of 86.31%. The median knowledge score was 5 out of 17. Lower monthly income (OR = 3.225, 95% CI = 0.255 to 2.141), higher number of comorbidities (OR = 2.724, 95% CI = 0.037 to 2.013), higher total knowledge score (OR = 1.334, 95% CI = 0.063 to 0.512) and respondent’s belief and attitude (OR = 0.813, 95% CI = -2.033 to -0.539), were found to be significantly associated with the frequency of lipid testing. In conclusion, the knowledge level regarding dyslipidaemia was below average and associated with a lower tendency to undergo frequent lipid testing. There is a need for innovative health awareness such as active educational campaigns in various settings of the community. Further qualitative studies that explore the understanding of the publics’, and antecedents of their, lipid screening behaviour are required. Appropriate communication by healthcare providers should be encouraged during patient consultations for higher impact.

## Introduction

Dyslipidaemia stands as a significant risk factor for numerous prevalent and life-threatening non-communicable diseases (NCDs), such as cardiovascular diseases (CVDs) [1, 2], diverse types of cancer [3–6], and diabetes [7, 8]. Global estimates revealed the widespread prevalence of hypercholesterolemia, with rates reaching 53.7% in Europe, 47.7% in America, 30.3% in Southeast Asia, and 23.1% in Africa [9].

Among Malaysian adults aged 30 years and above, a staggering 64% have been diagnosed with elevated total cholesterol (TC), while 56.7% exhibit elevated levels of low-density lipoprotein cholesterol (LDL-C), exceeding the Asian average by more than twofold [10].

Since dyslipidaemia is an asymptomatic condition, lipid screening is crucial in identifying at-risk individuals to prevent the development of symptomatic vascular diseases due to severe and untreated dyslipidaemia [11]. The United States Preventive Services Task Force (USPSTF) recommended lipid screening for adults aged 40 to 75 years, who have a 10-year cardiovascular risk exceeding 10% [12]. Correspondingly, in Malaysia, the Malaysian Clinical Practice Guidelines of 2017 mandates annual lipid profile assessments for all adults aged 30 and above [13].

Lipid screening practices are influenced by various factors globally. These factors include age [14], gender [15] educational level [16] ethnicity [17] education attainment [14, 18, 19] fear of abnormal test results and screening procedures [20] fear of higher healthcare costs associated with abnormal test results [14] fatalistic attitudes [14] income level [14, 15, 17] presence of underlying morbidities [14] absence of screening advice from healthcare personnel and perceptions of good health [14, 19] and lengthy waiting times at healthcare facilities [21].

Adequate knowledge in terms of the risk factors, screening availability and guidelines, and dietary requirements plays a crucial role in reducing the burden of non-communicable diseases (NCDs). Studies have shown the positive impact of knowledge on individuals’ screening practice. Norwegian women who were familiar with the recommended cervical screening interval were more likely to attend the screening [22].

Similarly, having underlying knowledge about the risk factors associated with diabetes was identified as a motivating factor for attending regular diabetes health screenings [21]. Patients who possessed knowledge about hypertension-related complications were more likely to undergo blood pressure screening [23]. Nonetheless, poor knowledge among women in Lagos, Nigeria, regarding the symptoms and risk factors of cervical cancer resulted in a low uptake of Pap smear tests [24]. Inadequate knowledge was found to be a deterrent to undergo colorectal screening [25].

Nevertheless, it is worth noting that certain studies have indicated a lack of a significant relationship between knowledge and the participation in health screenings [26, 27].

Due to inconsistent findings about knowledge and preventive health behaviours, and the scarcity of studies that specifically explore individuals’ knowledge regarding dyslipidaemia among the Malaysian population, we conducted this study to assess the association between the level of knowledge about dyslipidaemia and the frequency of lipid testing. In addition, we assessed the potential impact of predisposing, enabling, and need factors on the uptake of blood lipid screening within the Malaysian population.

## Materials and methods

### Procedure

This cross-sectional survey involved 314 Malaysian adults aged 30 and above who reside in Taman Selatan, Klang, Malaysia. The data collection process was carried out from 1st September 2022 to 31st October 2022. To participate in this study, participants were required to have the ability to read and comprehend either English or Malay language. Individuals diagnosed with conditions such as cognitive or psychiatric disorders, deafness, and blindness, were excluded from participating in the study.

The study adhered to the ethical guidelines set forth by the World Medical Association’s Helsinki Declaration for Human Studies. The study was approved by the International Medical University ethics committee with reference number 4.8/JCM-249/2022. Verbal informed consent was obtained from all respondents prior to their participation in the study. A representative from local community council had witnessed the data collection process and the verbal informed consent acquisition.

### Sample size determination

The minimum number of participants to be included in the study was calculated based on the formula derived from the work of Peduzzi et al. [28]. According to this formula, a minimum of 10 events per predictor variable is recommended. Considering that there were 10 independent variables related to predisposing, enabling, and need factors assessed in this study, with an assumed screening rate (*p*) set at 0.35, the sample size was determined to be 286. The assumed screening rate was derived from a similar study conducted by Alahmari et al. [18] wherein 34.5% of their study respondents reported undergoing lipid testing within a year. To accommodate a 10% non-response rate, the final sample size was calculated to be 314 participants.

### Sampling method

**Fig 1:**
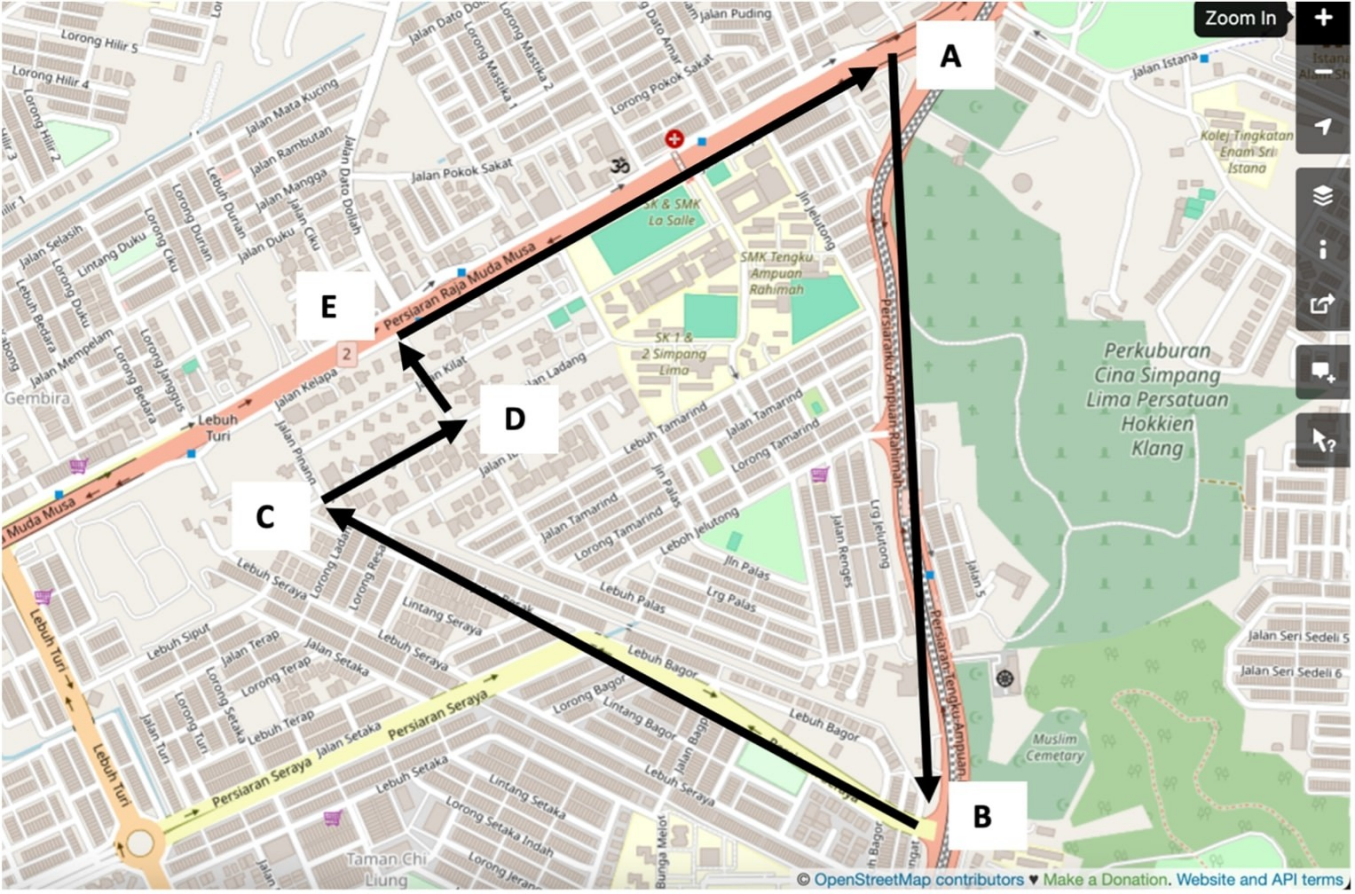
Map of Taman Selatan, Klang (source:www.openstreetmap.org).

The study was conducted in Taman Selatan, Klang, Selangor, Malaysia, which is home to a diverse group of residents from various multi-ethnic backgrounds and age groups. This residential area comprises low, middle, and high-income landed housing under freehold tenure, and is conveniently located near amenities like stores, parks, healthcare facilities, educational institutions, and places of worship. The neighbourhood is also well-connected via major highways, commuter rail systems, and light rail transits, ensuring easy accessibility for residents and visitors. The map of the study area is depicted in Fig 1.

Systematic random sampling was employed in this study, with landed homes being sampled at a fixed interval. To overcome constraints such as time and logistics, a modified sampling technique was adopted to calculate the sampling interval. The sampling interval was determined by dividing the sample size (N = 314 individuals) by the approximate number of landed homes to be interviewed daily (n = 25), resulting in an interval of 13.

The sampling process began from the outermost boundaries, moving inwards, specifically from Point A to Point E. The goal was to secure a minimum of 2 respondents from each household, strategically chosen to expedite reaching the desired sample size of 314. This approach was adopted to mitigate the potential of a lower response rate, ensuring a more reliable and timely achievement of the targeted sample size compared to interviewing just one respondent per household.

The study utilized computer software to randomly select the first landed home for the survey. Subsequently, the second target house was determined by adding the sampling interval to the number of the first site. This process was repeated until the desired sample size was attained.

If a house was found to be unoccupied at the time of visit, that house was revisited later that day or on another day. If a house was determined to be permanently vacant, or if the occupant’s declined participation, did not meet the inclusion criteria, or if an adult representative was unavailable after three separate visits, the next immediate household was approached as a substitute.

### Data collection instrument

To assess healthcare utilization in relation to lipid screening uptake, the Andersen behavioural model was utilized. This model is widely used to predict and explain individual choice and use of health services, as it provides a comprehensive guideline for conceptualizing the relationships between multiple explanatory factors, namely predisposing (demographic, social structure, health beliefs), enabling (personal, family, community) and need factors (perceived, evaluated) associated with utilization of health services [29].

The paper-based bilingual questionnaire consisted of 42 items and was developed in both English and Malay languages. Its design was based on a review of open-access journal articles, clinical practice guidelines, and information from official portals under the governance of the Ministry of Health Malaysia. Additionally, existing literature on the topic was considered during the questionnaire development process. The questionnaire comprised of four sections, which covered the following:

Section A: Consisted of 12 questions aimed at gathering sociodemographic information from the respondents. These questions explored factors such as age, gender, ethnicity, education level, occupation, income, smoking status, exercise frequency, presence of underlying health conditions, medication usage and frequency of lipid testing in the past year, with respondents indicating their lipid testing frequency as never done, once, twice, or more than twice.

Section B: Comprised of 17 closed-ended questions with response options of "Yes," "No," or "Not Sure" that assessed the respondents’ level of dyslipidaemia knowledge, in terms of risk factors associated with dyslipidaemia, dyslipidaemia guidelines and screening availability, and dyslipidaemia dietary knowledge. The total score for this section was calculated by summing the total number of correct responses which carries 1 mark, with a total attainable score of 17. The questions were formulated based on information obtained from the 5th edition of the Malaysian Clinical Practice Guideline on the Management of Dyslipidaemia (2017) [13] and the Clinical Practice Guidelines of Primary and Secondary Prevention of Cardiovascular Disease 2017 [30]. Additionally, the design of the dietary knowledge section was informed by a reliable and validated dyslipidaemia dietary knowledge questionnaire (DDKQ) developed by Liang et al. [31].

Section C: Consisted of seven 5-point Likert scale questions that assessed respondents’ beliefs and attitudes towards screening, in terms of fatalistic beliefs, perception of good health, fear of abnormal test results, screening procedure, and cost. Each response was assigned a point value, whereby the values for the options begin with “strongly agree” at 5, followed by “agree” at 4, “neutral” at 3, “disagree” at 2, and “strongly disagree” at 1 point respectively. The questions were adapted from a validated tool developed by Straughan and Seow [32] a local qualitative study [19] and data obtained from the 2010 National Health Survey conducted in Singapore [14].

Section D: Evaluated the issues faced by respondents with regards to healthcare facilities and healthcare professionals that might hinder their ability to undergo lipid testing. The design of this section was based on a local qualitative study that focused on the experiences reported by post-menopausal women residing in the East Coast of Malaysia in relation to cardiovascular screening [19]. The questionnaire was pilot tested among 15 participants to gauge respondent understanding and internal consistency of questionnaire’s items.

### Reliability of data collection instrument

Cronbach’s alpha obtained from pilot study for questions that assessed the total knowledge of respondents about dyslipidaemia, in terms of associated risk factors, guidelines, screening availability, and dietary intake, the Cronbach alpha reliability coefficient was 0.919 (for a total of 17 items), indicating a very good level of reliability. Similarly, for questions that assessed respondents’ beliefs and attitudes towards screening, including predisposing factors such as fatalistic attitudes, perception of good health, and fear, the Cronbach alpha was 0.822 (for a total of 7 items), also falling within a very good level of reliability. However, the questions that assessed healthcare-related factors (need factors) that might have impacted lipid testing frequency showed a Cronbach alpha reliability coefficient of 0.659 (for a total of 5 items), which fell below an acceptable level of reliability [33].

### Statistical analyses

All data were entered and analysed using SPSS Software Version 27. The median and interquartile range of the total knowledge score was calculated. Bivariate analysis and multivariable ordinal regression were conducted to assess the strength of the relationship between the predisposing, enabling and need factors with the frequency of lipid testing. All variables irrelative of their significance from bivariate analysis were included in the multivariable ordinal regression to allow the reader to appreciate the confounding variables if any. The significance level was set at 0.05.

## Results

The response rate of this study was 86.31% with 271 total respondents. According to Table 1, among the adults surveyed, the highest proportions were observed for female respondents (53.9%), aged 40–49 (29.9%), of Indian ethnicity (53.1%), attained secondary school education (58.7%), employed (72.7%), has a monthly income ranging from RM1 to RM4851 (73.8%) and were non-smokers (64.9%). Around 42.1% of the respondents reported having at least one of the eight morbidities associated with dyslipidaemia, while 39.5% of the respondents reported taking medications for at least one of the eight morbidities mentioned in the questionnaire. Within the past year, 53.5% of respondents had conducted lipid testing once.

**Table 1:** Baseline characteristic of respondents.

Variables		n	%
Age	30–39	68	25.1
	40–49	81	29.9
	50–59	55	20.3
	60–69	67	24.7
Gender	Male	125	46.1
	Female	146	53.9
Ethnicity	Malay	67	24.7
	Chinese	60	22.1
	Indian	144	53.1
	Others	0	0
Education	No formal education	1	0.4
	Primary	4	1.5
	Secondary	159	58.7
	College/University	107	39.5
Occupation	Unemployed/Retired	58	21.4
	Housewife	16	5.9
	Student	0	0
	Employed	197	72.7
Income (MYR^*+*^)	No income	36	13.3
	MYR1 –MYR4,850	200	73.8
	MYR4,851 –MYR10,970	34	12.5
	>MYR10,970	1	0.4
Smoking status	Never Smoked	176	64.9
	Ex-smoker	76	28.0
	Current Smoker	19	7.0
No. of comorbidities per respondent	0	157	57.9
	1	40	14.8
	2	36	13.3
	3	34	12.5
	4	4	1.5
No. of medication taken per respondent	0	164	60.5
	1	39	14.4
	2	31	11.4
	3	36	13.3
	4	1	0.4
Lipid testing frequency	Never done	107	39.5
	Once	145	53.5
	More than once	19	7.0
Age	Median (IQR)	49 (20)	

^+^MYR = Malaysian Ringgit (The currency used in Malaysia)

### Knowledge about dyslipidaemia

Table 2 displays the distribution of correct answers to questions assessing respondents’ knowledge regarding the risk factors, screening availability and guidelines, and dietary requirements associated with dyslipidaemia, as well as the median and interquartile range. Overall, most respondents displayed a less than average knowledge score for dyslipidaemia, scoring 5 out of 17.

**Table 2:** Distribution of answers to knowledge questions.

Domain	Questions	% Correct Answer	Total possible score	Median (IQR)
**Knowledge about risk factors associated with dyslipidaemia**	Low physical activity leads to dyslipidemia	93.0	7	1(0)
	Diabetes mellitus leads to dyslipidemia	1.8		
	Obesity leads to dyslipidemia	3.0		
	Dyslipidemia may be inherited from parents	1.1		
	Dyslipidemia leads to heart attack	1.1		
	Dyslipidemia leads to plaque build-up on arterial walls	1.1		
	Dyslipidemia leads to fatty liver	2.6		
**Knowledge regarding screening guidelines and availability**	The Ministry of Health Malaysia has recommended that all people who are at risk of getting dyslipidaemia to carry out blood lipid test once a year	1.1	4	2(0)
	Blood lipid test is available in most private clinics	91.1		
	Blood lipid test is available in most government clinics	93.4		
	Blood lipid test is free for eligible low-income individuals (monthly household income less than RM4,850)	1.1		
**Dietary knowledge**	Foods such as vegetables, fruits, legumes, and whole grain cereals can help maintain normal blood lipid level	96.3	6	2(1)
	Vegetable oil, fatty fish and nuts are examples of food that are low in cholesterol	0.4		
	Individuals with dyslipidemia should minimize the consumption of processed meat	52.4		
	Individuals with dyslipidemia should minimize the consumption of bakery products	10.3		
	High intake of coconut oil, palm kernel oil and palm oil may increase the likelihood of dyslipidemia	4.4		
	High intake of beef, pork, milk, yogurt, and cheese may decrease the likelihood of dyslipidemia	8.1		
	**Total Knowledge Score**		**17**	**5(1)**

Regarding risk factors, 93% respondents were aware that low physical activity can lead to dyslipidaemia but were unaware of the associated chronic diseases such as diabetes mellitus, obesity, heart attack, atherosclerosis, and fatty liver. Among screening guidelines and testing availability, 91.1% and 93.4% of respondents were aware that blood lipid test was available in private and government clinics respectively. However, only 1.1% of the respondents were aware that the Ministry of Health Malaysia recommends lipid screening at least once a year for those at risk and that lipid testing is free for those with low monthly income. In terms of dietary knowledge, 96.3% of respondents were aware that fruits, vegetables, legumes, and whole grain cereals help to maintain normal lipid levels but were unaware of examples of food low in cholesterol and types of food that may increase likelihood of dyslipidaemia.

### Personal beliefs and attitudes, and impact of healthcare factors on lipid testing practices

Table 3 depicts the distribution of answers pertaining to the respondents’ beliefs and attitudes (predisposing factors) regarding the adoption of lipid testing. The majority of respondents did not have fatalistic views towards dyslipidaemia, with 70.5% disagreeing that dyslipidaemia is inevitable and cannot be reversed and 76.0% agreeing that it is preventable. Although 47.2% disagreed that lipid testing should not be done if healthy, a considerable proportion (50.2%) remained undecided about requesting a test while being healthy. Over 50% of the respondents were unsure about their responses to the fears of lipid testing, including anxiety over results, painful procedures, and costly test.

**Table 3:** Distribution of respondents’ beliefs and attitudes, and impact of healthcare factors on lipid testing practices.

Domain			SD	D	N	A	SA
**Belief and Attitude**	Fatalistic Behaviour	If dyslipidaemia is fated to happen, nothing can be done to change it	35 (12.9)	156 (57.6)	78 (28.8)	0 (0)	2 (0.7)
		Dyslipidaemia can be prevented, it’s up to me to do something about it	0 (0)	2 (0.7)	63 (23.2)	176 (64.9)	30 (11.1)
		If dyslipidaemia was meant to occur, there’s no point in doing blood lipid tests	26 (9.6)	116 (42.8)	126 (46.5)	2 (0.7)	1 (0.4)
	Perception of Good Health	If I feel healthy, I should not do a blood lipid test	18 (6.6)	110 (40.6)	136 (50.2)	4 (1.5)	3 (1.1)
	Fear	Knowing the results of the blood lipid test may cause unnecessary worry	17 (6.3)	96 (35.4)	151 (55.7)	5 (1.8)	2 (0.7)
		Blood lipid testing is a painful procedure	5 (1.8)	68 (25.1)	197 (72.7)	0 (0)	1 (0.4)
		Blood lipid tests are costly	3 (1.1)	66 (24.4)	199 (73.4)	3 (1.1)	0 (0)
**Health-care Reasons**	Dyslipidaemia has no visible symptoms requiring treatment		0 (0)	3 (1.1)	260 (95.9)	8 (3)	0 (0)
	Healthcare centres have long waiting times for blood lipid testing		0 (0)	17 (6.3)	203 (74.9)	48 (17.7)	3 (1.1)
	Healthcare providers’ information on blood lipid testing availability aids in dyslipidaemia prevention		0 (0)	0 (0)	66 (24.4)	200 (73.8)	5 (1.8)
	Healthcare staff should advise on blood lipid testing		0 (0)	0 (0)	50 (18.5)	216 (79.7)	5 (1.8)
	Requesting a blood lipid test is inappropriate for healthy individuals		3 (1.1)	45 (16.6)	209 (77.1)	11 (4.1)	3 (1.1)

*SD = Strongly Disagree, D = Disagree, N = Neutral, A = Agree, SA = Strongly Agree

Attitude toward the potential influence of healthcare factors (need factors) on lipid testing practices is shown in Table 3. Over 70% of the respondents were uncertain about the symptoms of dyslipidaemia and waiting times at healthcare centers for lipid testing. However, over 70% of respondents agreed that healthcare providers should offer advice on lipid testing and provide information on lipid testing availability.

### Factors associated with frequent lipid testing

Based on Table 4, it is evident that individuals with low monthly income had higher odds of undergoing more frequent lipid testing, (OR = 3.225, 95% CI = 0.255 to 2.141) than those in the higher income group. Furthermore, individuals with a greater number of comorbidities exhibited increased odds of frequent lipid testing (OR = 2.724, 95% CI = 0.037 to 2.013), as compared to those with a lower number of comorbidities. Additionally, participants with a higher total knowledge score were 1.334 times more likely to undergo frequent lipid testing (OR = 1.334, 95% CI = 0.063 to 0.512) than their counterparts with a lower total knowledge score. Lastly, the odds of frequent lipid testing were 0.813 times lower among individuals who exhibited higher fatalistic attitudes, fear, and a perception of good health (OR = 0.813, 95% CI = -2.033 to -0.539) than among those who exhibit an optimistic outlook, courage, and a poor perception of health.

**Table 4:** Factors associated with frequency of lipid testing.

Variables^*+*^		OR	*p*	95% Confidence Interval	
				Lower Bound	Upper Bound
Age		0.998	0.915	-0.041	0.037
Sex	Male	1.310	0.423	-0.391	0.931
	Female	1	1	1	1
Ethnicity	Malay	0.764	0.455	-0.937	0.420
	Chinese	0.503	0.056	-1.430	0.019
	Indian	1	1	1	1
Education	Secondary Education and Lower	1.473	0.251	-0.275	1.051
	College/University Education	1	1	1	1
Occupation	Unemployed/retired	1.188	0.685	-0.805	1.226
	Housewife	0.660	0.583	-1.706	0.960
	Employed	1	1	1	1
Income (MYR^*+*^*)*	0–4850	3.225	0.013*	0.255	2.141
	>4851	1	1	1	1
Frequency of Exercise		1.151	0.288	-0.117	0.393
Smoking status	Never Smoked	2.440	0.201	-0.454	2.157
	Ex-smoker	2.012	0.327	-0.640	1.922
	Current Smoker	1	1	1	1
Number of Comorbidities per Respondent		2.724	0.042*	0.037	2.013
Number of Medications Consumed per Respondent		0.838	0.699	-1.248	0.837
Total Knowledge Score		1.334	0.012*	0.063	0.512
Belief and Attitude		0.813	0.001*	-2.033	-0.539
Healthcare Reasons		0.634	0.495	-1.705	0.824

^+^Dependent variable: Frequency of lipid testing

## Discussion

The relationship between knowledge level and adoption of specific health behaviour is documented in the literature. We hypothesized that knowledge level is a key determinant of lipid testing behaviour. The results of this study show that the knowledge level regarding dyslipidaemia was less than average. Higher knowledge levels, lower income, concurrent comorbidities, and fatalistic attitude were associated with lipid testing frequency.

This knowledge deficit could potentially be attributed to a lack of awareness about dyslipidaemia, primarily because it is an asymptomatic condition that may not capture individuals’ attention. Furthermore, the absence of adequate health education might also contribute to the respondents’ lack of awareness regarding dyslipidaemia. Studies have emphasized that the inclusion of health promotion education related to chronic noncommunicable diseases plays a crucial role in enhancing knowledge and attitudes related to health-promoting behaviours, ultimately aiding in the prevention of these diseases [34]. It is also possible that the respondents may have experienced poor health literacy and communication obstacles, resulting in limited awareness regarding dyslipidaemia. For instance, when patients and healthcare professionals do not share a common language, essential information regarding dyslipidaemia, including its risk factors, dietary requirements, and screening guidelines may not be effectively conveyed [13]. Our results are in line with those reported by a Saudi Arabia cohort of teachers [35] and a multiethnic Asian population in Singapore [17] who showed poor knowledge about dyslipidaemia.

The findings of this study highlight a positive correlation between knowledge levels and the frequency of lipid testing. The association between a low knowledge level and dyslipidaemia can be attributed to the fact that individuals lacking sufficient knowledge may struggle to make appropriate dietary choices, adopt a healthy lifestyle, assess the risk of such silent diseases, and may not participate in screening programs. These assumptions were supported by our results. Notably, respondents demonstrated a below-average level of knowledge concerning dyslipidaemia, its risk factors, guidelines, test availability, and dietary requirements, as evidenced by the low proportion of correct answers to most questions.

Other studies have reported similar findings for instance poor knowledge regarding dyslipidaemia is associated with poor adherence to preventive behaviours to manage the disease [18, 36, 37]. On the same note, our respondents’ belief that healthcare staff should offer guidance on blood lipid testing, is likely influenced by the perception that doctors are the primary source of health information. This observation aligns with prior research emphasizing the pivotal role of physician recommendations in promoting health screening uptake [25, 38], and their status as the primary source of medical information [18, 39].

The negative association between heightened fear and stringent fatalistic attitudes and lipid testing frequency aligns with existing literature, in which, fear of abnormal test results [21] screening procedure [40], and cost [14, 21] were found to be significant barriers to medical screenings, as these factors sway an individual’s health and happiness in a negative direction [14, 41]. This result may be explained by anxiety about diagnoses, a lack of adequate knowledge, the existence of misinformation, and a lack of trust in the healthcare system. Anxiety is an inevitable attribute that arises when facing uncertainties. It is intuitive for an individual to fear the consequences of specific test procedures [42] and diagnoses [43]. Nonetheless, sufficient knowledge about the test and disease, which is lacking among our sample, would reduce such anxiety. Distrust in healthcare professionals and hospitals negatively impacts patients’ decisions and outcomes. This issue is exemplified when patients have no choice in selecting their healthcare provider [44].

On the other hand, the negative influence of positive perception of one’s health on the uptake of lipid tests in our study could be attributed to the widespread misconception that health screenings are only necessary when one is already experiencing illness and a lack of awareness regarding the prophylactic role of health screenings [21].

In addition to the level of knowledge, our investigation unveiled an association between monthly income and the frequency of lipid testing. Respondents, with lower monthly incomes were more likely to undergo frequent testing. Interestingly, this finding diverges from existing literature, which suggests that individuals with higher incomes tend to participate in more frequent screening activities [14, 15, 45, 46]. This could be explained by the fact that individuals with lower incomes may have a higher prevalence of comorbidities, making them more health-conscious. Respondents of our studies with low income exhibited a higher proportion of comorbidities. Moreover, the affordability of attending government clinics might have contributed to this phenomenon. Low income, often associated with lower employment, may result in more free time, which could lead some to visit health facilities more frequently. Low-income communities are usually targeted by non-governmental organizations (NGOs) and health promotion campaigns by educational institutions and healthcare organizations.

Respondents with higher comorbidities had higher odds of frequent lipid testing. This is in line with existing literature showing that individuals with comorbidities, such as hypertension or diabetes mellitus, are more inclined to undergo screenings for other chronic diseases [14]. The higher tendency to undergo frequent testing might be explained by the increased health consciousness caused by the risk of concurrent disease. It is also likely that such individuals got advice from health professionals during the follow-up of their comorbidities. A report showed that colorectal screening rates were high among veterans with moderate and high comorbidities [47].

The study findings draw some public health implications that stakeholders could utilize. It highlights the importance of health promotion campaigns to increase awareness about the risk of dyslipidaemia and the necessity of regular screening. It also highlights the importance of patient empowerment to shift the responsibility from healthcare providers to patients themselves. Doctor-patient communication should be emphasized as an effective method of delivering crucial health information. The role of knowledge deficit may highlight limited access to information by some demographic groups.

This study acknowledges several limitations. First, despite the community composition resembling a Malaysian structure, data from a single locality may limit generalizing the results to a larger Malaysian population. The adoption of the random sampling method ensured representativeness and supported generalizing the results to the target population. Second, the accuracy of results obtained with a self-administered questionnaire is typically influenced by the literacy level of the participants. The authors followed a sound methodological approach in ensuring the clarity and validity of the questionnaire to minimize the possible effect of respondent literacy on understanding the survey. Nonetheless, none of the mentioned limitations seem to have affected the obtained results or interpretation.

## Conclusion

The findings of the study elucidate that the knowledge level regarding dyslipidaemia was below average and associated with a lower tendency to undergo frequent lipid testing. This highlights the need for innovative health awareness campaigns. This may include proactive measures, such as active educational campaigns in various settings of the community. To guide policymakers in designing effective health promotion interventions for dyslipidaemia, further qualitative studies that explore the understanding of the public and antecedents of their lipid screening behaviour are required. This may include a more diverse sample to outline additional motivators and barriers to lipid screening. Appropriate communication by healthcare providers should be encouraged during patient consultations for higher impact.

## Supporting information

S1 File Pilot study dataset. (SAV)

S2 File Actual study dataset. (SAV)
